# Extraction and biomolecular analysis of dermal interstitial fluid collected with hollow microneedles

**DOI:** 10.1038/s42003-018-0170-z

**Published:** 2018-10-22

**Authors:** Philip R. Miller, Robert M. Taylor, Bao Quoc Tran, Gabrielle Boyd, Trevor Glaros, Victor H. Chavez, Raga Krishnakumar, Anupama Sinha, Kunal Poorey, Kelly P. Williams, Steven S. Branda, Justin T. Baca, Ronen Polsky

**Affiliations:** 1 Nano and Micro Sensors, Sandia National Laboratories, Albuquerque, NM 87185 USA; 2 Department of Emergency Medicine, The University of New Mexico, Albuquerque, NM 87131 USA; 3 Excet Inc., 6225 Brandon Ave, Suite 360, Springfield, VA 22150 USA; 4 Research and Technology Directorate, US Army Edgewood Chemical Biological Center, Aberdeen Proving Ground, Edgewood, MD 21010 USA; 5 Systems Biology, Sandia National Laboratories, Livermore, CA 94551 USA; 6 Biomass Science and Conversion Technology, Sandia National Laboratories, Livermore, CA 94551 USA

## Abstract

Dermal interstitial fluid (ISF) is an underutilized information-rich biofluid potentially useful in health status monitoring applications whose contents remain challenging to characterize. Here, we present a facile microneedle approach for dermal ISF extraction with minimal pain and no blistering for human subjects and rats. Extracted ISF volumes were sufficient for determining transcriptome, and proteome signatures. We noted similar profiles in ISF, serum, and plasma samples, suggesting that ISF can be a proxy for direct blood sampling. Dynamic changes in RNA-seq were recorded in ISF from induced hypoxia conditions. Finally, we report the first isolation and characterization, to our knowledge, of exosomes from dermal ISF. The ISF exosome concentration is 12–13 times more enriched when compared to plasma and serum and represents a previously unexplored biofluid for exosome isolation. This minimally invasive extraction approach can enable mechanistic studies of ISF and demonstrates the potential of ISF for real-time health monitoring applications.

## Abstract (web summary)

Philip Miller et al. present an approach for extracting dermal interstitial fluid (ISF) using an array of hollow microneedles in a cylindrical substrate that minimizes skin compression and tissue damage. They extract larger volumes of ISF suitable for downstream analyses, compared to previous reports.

## Introduction

Standard clinical testing typically involves collecting biological fluid samples such as blood, urine, sweat, saliva, and sputum for laboratory analysis^1^. With the growing need for non-invasive sampling and real-time physiological monitoring, interest in exploring the skin as a reservoir of information has grown in recent years^2–4^. Dermal interstitial fluid (ISF) holds promise as a potentially rich and accessible source of information, but minimally invasive collection of ISF has proved challenging. Additionally, there is a paucity of knowledge on the presence of useful physiological markers in ISF.

Numerous publications have attempted to elucidate the biomolecular content of dermal ISF without wide agreement on contents, particularly with respect to protein markers^5–9^. Many of these used extraction methods (i.e. suction blister, effusion, dialysis, or sonication) that alter the composition of ISF, due to the local trauma caused by the extraction process. For instance, the suction blister fluid (SBF) method likely causes extensive cell lysis, destabilization of the stratum corneum, and separation of the dermal layers^10^. Additionally, previously reported extraction methods do not appear to be compatible with practical real-time monitoring of physiological changes with the exception of glucose monitoring such as the GlucoWatch Biographer, which uses reverse iontophoresis to extract glucose from the skin.

Microneedle-enabled ISF extraction has been proposed for minimally invasive monitoring and diagnostic applications^11^. While microneedles provide very precise skin penetration, extraction of sufficient ISF (10–20 µl) for transcriptomic or proteomic analysis has not been reported^12–14^ (http://www.rsc.org/images/LOC/2015/PDFs/Papers/0912_W.201c.pdf)^15^. Herein, we report a facile method that uses an array of hollow microneedles to extract large quantities (up to 20 µl and 60 microliters from humans and rats, respectively) of dermal ISF, with no need for blistering of the skin and uses widely available materials for easy construction. We extract ISF volumes sufficient for common downstream analyses, such as transcriptomic and proteomic profiling, and exosome isolation. Exosomes are increasingly being shown to be effective for liquid biopsy applications, however, their isolation from blood is difficult due to its complicated matrix making ISF an intriguing substitute. Additionally, we find that dermal ISF possesses transcriptomic and proteomic content highly similar to serum and plasma. ISF could therefore constitute an informative proxy for blood in health monitoring, and microneedle-enabled sampling could advance wearable, real-time sensing devices.

## Results

### ISF extraction mechanism

Microneedle insertion and ISF extraction is complicated by the dynamic properties and elasticity of skin. Stretching and tenting of skin impedes the placement of single and arrayed microneedles^16,17^. After skin puncture, dermal compaction around the microneedle insertion site is believed to increase fluidic resistance in drug delivery studies. Wang et al. showed that reducing the amount of dermal compaction results in higher flow rates during drug delivery^18^. Many groups have attempted ISF collection with microneedles, but limited volumes (<2 µl) were collected, limiting characterization and analysis^12–14^ (http://www.rsc.org/images/LOC/2015/PDFs/Papers/0912_W.201c.pdf)^19,49^. We present a needle substrate that minimize dermal compaction at insertion site(s), allowing extraction of higher ISF volumes.

**Fig. 1:**
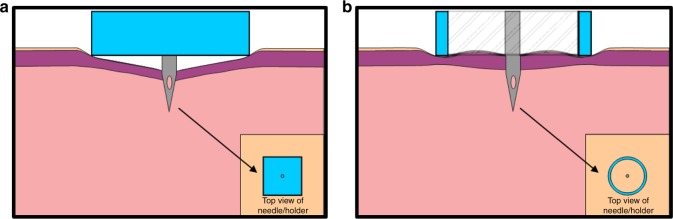
Microneedle configurations. Planar (**a**) and cylinder concentric (**b**) microneedle substrates

Traditional needle substrates are almost exclusively flat planar projections (Fig. 1a). We believe this planar arrangement causes tissue compaction and displacement of ISF from the skin, which can contain 15–40% ISF by volume depending on the layer^20^. To test this hypothesis, we created an alternative needle substrate that creates a concentric opening about the microneedle (Fig. 1b), such that the skin surrounding the needle was not compressed and that also results in an inherent pumping mechanism for ISF extraction.

### Microneedle design and fabrication

**Fig. 2:**
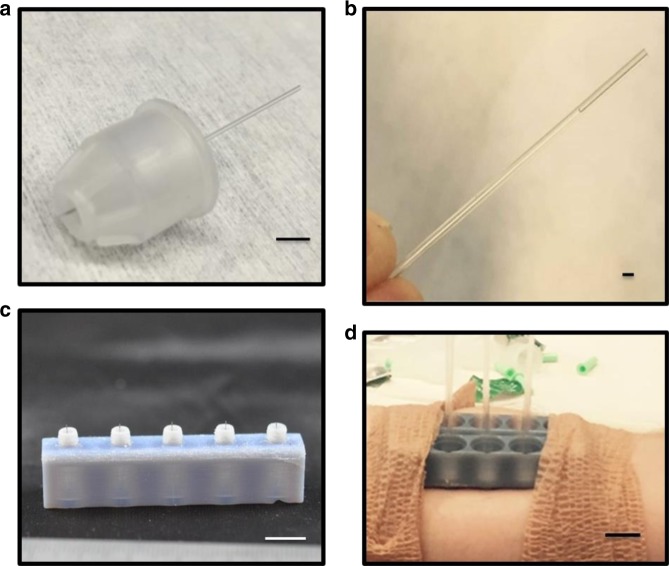
Microneedle assembly and arrays. **a** Single microneedle (length of 1500 µm) within polymer housing with glass capillary collection tube. **b** Glass capillary collection tube with ISF collected from a human subject using a 1500 µm microneedle length. **c** 3D printed microneedle holder for 1500 µm needle length; inner diameter of concentric ring surrounding microneedles are ~2.8 mm. **d** Two 3D microneedle holders adhered to a human subject for ISF extraction and collection in glass capillary tubes. Scale bars; (**a**): 5 mm, (**b**): 1 mm, (**c**): 1 cm, (**d**): 1 cm

Microneedles were created from Single BD 32 G 4 mm Ultra-Fine Pen needles (Becton Dickinson) using a CO_2_ laser cutter with a three-axis stage to cut the protective plastic housing and precisely control needle lengths (Supplementary Figure 1). The cutting of the housing produces a concentric opening around the microneedle when pressed to skin. A glass capillary was then attached to the backing of the microneedle (Fig. 2a) for ISF collection. After application a 30–120 s lag time was observed for ISF flow to enter the capillary that would continue for 10–15 min, on average. Figure 2b shows collected ISF inside the capillary after removal from the microneedle. The extracted fluid was colorless (no red blood cells) while visible microscope analysis and electron microscopy staining showed no cellular components that would indicate the presence of excess tissue from insertion damage. It is important to note that the flow of fluid into the glass capillary is not attributed to capillary action. No wicking of liquid into the needle/capillary sets occurred when placed into deionized water control droplets. Abe et al. previously characterized the same commercial pen needle system, and noted that hydrophobicity of the inner bore was slightly decreased by cleaning with UV-ozone^21^, however in our experience, this cleaning step was not sufficient to induce capillary flow. Therefore, we attribute the ISF flow to the local pressure induced from ring edges at tissue surrounding the insertion point from the unique concentric microneedle opening geometry. In contrast to other methods used to collect dermal interstitial fluid (i.e. blister and dialysis), no separate instrumentation is required (i.e. vacuum pump).

Three different microneedle lengths (1000 µm, 1500 µm, and 2000 µm) were initially studied for their ability to extract fluid from a human forearm with minimal pain response. In this pilot study, ISF extraction was successful in four of seven human subjects. Fluid was extracted with each microneedle length, with 1500 µm needles eliciting a higher percentage of extraction success compared to the other lengths (31% vs. 14 and 16%, respectively); average total volume was 1.51 µl for single microneedles (*n* = 10). For each needle length, we recorded pain scores on insertion (pain scale of 0–10 with 0 indicating absence of pain). Scores of 0.0 ± 0.0, 0.21 ± 0.49, and 0.71 ± 1.11 were reported for the 1000 µm, 1500 µm, and 2000 µm microneedle lengths, respectively. A length of 1500 µm was therefore selected for subsequent studies of arrayed microneedles. Histology of excised rat skin to determine tissue penetration depths was attempted but ultimately inconclusive. The insulin delivery PEN needles used are pre-coated with a silicone lubricant and have an engineered five grind bevel tip geometry to reduce insertion penetration force, and limit pain response which likely contribute to the inability of effectively imaging the excised tissues for damage. We also believe that these same design properties, engineered to minimize tissue trauma, also preclude rupturing of capillaries which allowed extraction of ISF with no blood contamination for tested needle lengths up to 1500 µm.

Arrays were then fabricated to increase extraction volumes of ISF. Each array was 3D printed to contain up to five needles in parallel while maintaining the open concentric design at the base of each needle (Fig. 2c and d). Using the 5-microneedle arrays, up to 16 µl of ISF was extracted in 1–2-h periods in human subjects. This represents a 4–5 increase in extraction efficiency over previous attempts which report on average ~1 µl for 30 min of extraction^12–14^ (http://www.rsc.org/images/LOC/2015/PDFs/Papers/0912_W.201c.pdf)^15^. Overall the success rate of ISF extraction was 92.9%, and more than 10 µl was extracted in 64% of subjects (*n* = 14). No observed blockage of the needle pores was observed visually after removal, and needles remained open to fluid flow with syringe testing. Further arrangements of needle density and spacings have not been attempted.

Participants were asked to score the pain at array insertion, after placement for 5–10 min., and upon removal of the arrays. Scores of 1.16 ± 0.75, 0.56 ± 0.70, and 0.18 ± 0.27 were reported for the start, middle, and end of the procedure, respectively (*n* = 14). No bruising of the skin or other adverse events were witnessed by investigators or disclosed by participants.

### Identification and characterization of exosomes from ISF and serum

**Fig. 3:**
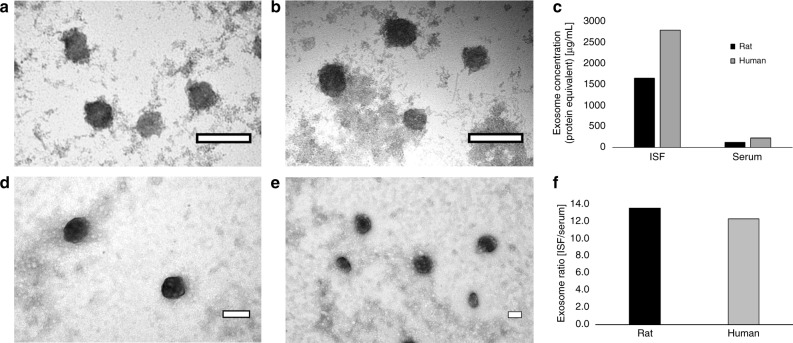
Rat and human characterization. TEM images of rat (**a**, **b**) and human (**d**, **e**) exosomes purified from ISF (**a**, **d**) and serum (**b**, **e**). Exosome concentration (protein equivalent) was measured using a nanoDrop spectrophotometer and is shown in (**c**). The exosome ratio of ISF vs. serum in rat and human samples is shown in (**f**). Scale bars; (**a**, **b**, **d**, **e**): 100 nm

Exosomes are recently described, cell-derived vesicles containing high concentrations of proteins and nucleic acid^22,23^. Exosomes appear to play important roles in intercellular signaling, and contain clinically relevant biomarkers for cancer and other illnesses^23–25^. We report here the first description to our knowledge of exosome content in dermal ISF, to our knowledge. Exosomes from rat and human ISF and serum were isolated using standard exosome isolation kits from Invitrogen and visualized using TEM (Fig. 3). Using ImageJ Software^25^, exosome diameters purified from rat and human ISF were 140.4 ± 74.8 nm and 101.2 ± 43.9 nm, respectively, while exosomes purified from rat and human serum had diameters of 105.5 ± 42.0 nm and 103.2 ± 37.2 nm, respectively. Measured exosomes diameters agree with those established in the literature (30–150 nm)^23–27^. Using a nanoDrop spectrophotometer, we quantified the protein equivalent of purified exosome preparations from the ISF and serum of rats and humans. We found that, in rats, there were 1643 µg per ml and 122 µg per ml protein equivalents of exosomes in ISF and serum, respectively (Fig. 3c). In a human subject, spectrophotometric analysis revealed that there were 2,786 µg per ml and 228 µg per ml protein equivalents of exosomes in ISF and serum, respectively (Fig. 3c). Interestingly, both rat and human ISF was found to be highly enriched for exosomes, with ISF having 12–13 times more exosomes (protein equivalents) than serum (Fig. 3f). The size of exosomes limits their ability to cross venous capillaries, thus they are logically surmised to be derived from local tissue mostly (the skin is the largest organ in the human body with a high concentration of immune cells). Slow diffusion rates in the high density of collagen matrix likely contribute to the enriched presence in ISF compared to plasma. Dermal ISF does not benefit from a pumping mechanism (like blood) that induces flow and its clearance is dependent on drainage into the lymphatic system, which should also introduce the exosomes into systemic circulation. Proteomic results (described below) support the conclusion of an enriched exosome presence in ISF. This points to exosomes in dermal ISF as a potentially rich source of biomolecular information previously unexplored.

### Proteomic analysis of dermal ISF vs. plasma and serum

**Fig. 4:**
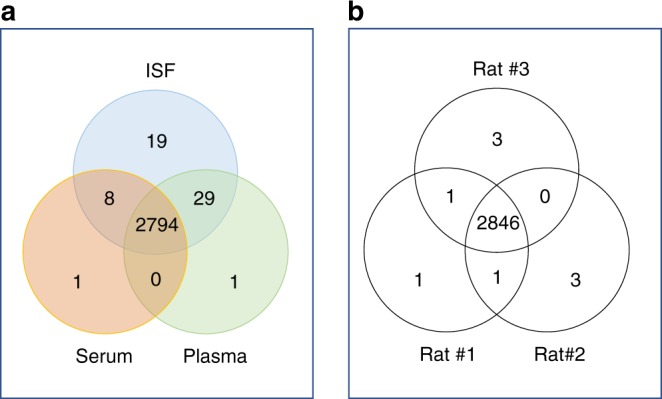
Sample overlap. **a** Venn diagram demonstrating distribution of 2852 proteins identified in plasma, serum and ISF of rat #1. **b** Complete ISF proteome distribution of rats 1, 2 and 3, with 99.9% proteins were consistently identified in all three rats

Qualitative and quantitative proteomic comparison of ISF with plasma and serum was studied on three rats by combining a two-dimensional LC/MS analysis with a stable heavy isotopic labeling technique (TMT tags). The analysis resulted in the identification of 1976 protein groups containing a total of 2855 individual proteins. Nearly 100% of the proteins identified in the blood-derived samples of rat #1 were also detected in ISF, and just a couple dozen other proteins (<1%) were specific to ISF as depicted in Fig. 4a. Similar results were observed for two other examined rats (Supplementary Figure 2). Reproducibility of the ISF protein identifications was extremely high for the small sample size used, as demonstrated by the fact that 99.9% proteins were found to be in common among all three rats examined (Fig. 4b).

Nearly all proteins detected in the ISF were also detected in serum and plasma, suggesting that ISF-unique proteins previously detected through profiling of blister-derived ISF may be related to tissue damage or inflammatory response (although a direct comparison to SBF was not done in this study). In a previously reported proteomic study of SBF, Kool et al. demonstrated the content of human SBF was more diverse than serum, with 46% of the 442 proteins identified unique to SBF^27^. Given our findings, the diversity of SBF proteome could be attributed to protein leakage from tissue proximal to the blister site, due to tissue damage caused by the vacuum force applied during sampling. This also suggests that the ISF collected in our technique is a purer filtrate of plasma. Proteomics on plasma and serum is particularly challenging due to the large dynamic range of proteins within the blood which makes the sampling in ISF more attractive for clinical diagnosis.

**Fig. 5:**
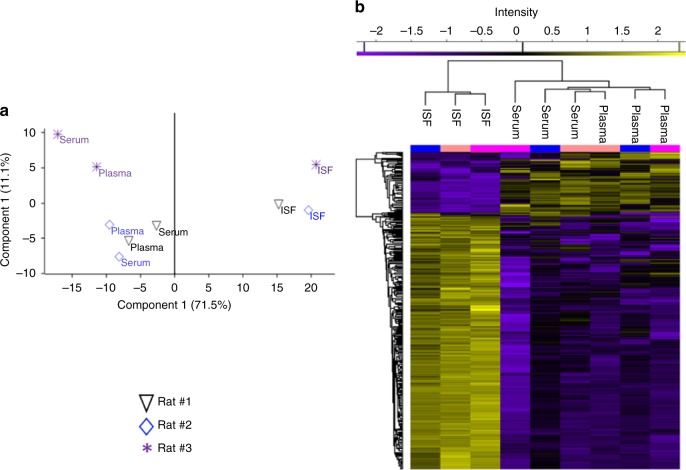
**a** Protein quantification and clustering. ANOVA plot demonstrating the statistical difference of 862 proteins quantified in plasma, serum and ISF of three examined rats (permutation-based FDR 0.05). **b** Two-dimensional, *Z*-score based hierarchical clustering plot of 330 proteins with a minimum expression ratio of twofold in all three rats. Higher expression is red and lower expression is green

Although the qualitative analysis was identical between ISF proteins and those of plasma and serum, the relative abundance expression of proteins in these fluid types were highly distinctive. A stringent criterion was applied for quantitation, which resulted in 862 proteins that were unambiguously identified and had valid counts of reporter ion intensity in all three replicates of at least one of the three fluids. Analysis of variance (ANOVA) of these proteins identified 560 proteins with notable differences in abundance, and ISF was clearly distinguishable from serum and plasma (Fig. 5a). Among the ANOVA significant proteins, 330 proteins (38%) belonged to ISF and were detected at a (+/−) two fold minimum difference in abundance relative to levels in plasma or serum; 29 of these proteins were detected at lower levels in ISF, and 301 at higher levels in ISF (Fig. 5b). A large portion (70%) of the proteins that were detected at higher levels in ISF are known to be associated with the exosome, as determined by annotation enrichment analysis (Supplementary Table 1). This observation is consistent with the finding that exosomes were 12–13 times more concentrated in ISF than in serum, as seen in the TEM imaging and exosome concentration measurement section presented in Fig. 3.

In summary, we find consistent signatures of protein abundance in ISF. The ISF proteome is essentially identical to the serum and plasma proteomes with regard to constituent proteins detected, but characteristically different with regard to the relative abundances of those proteins. The ISF also contains higher concentrations of exosomes than detected in serum and plasma.

### Transcriptomic profiles of dermal ISF, serum, and plasma

Dermal ISF, serum, and plasma matching samples were collected from four rats; additionally, a second plasma sample was collected from three of the four rats, to enable comparison of duplicate samples versus different sample types from the same rat by Next Generation Sequencing (NGS). As shown in Table 1, the majority of potentially informative RNA species detected in all sample types were transcripts derived from protein-coding genes (CDS), which on average accounted for 63.2% to 78.9% of all RNA species detected in the samples. The next most abundant RNA species was long non-coding RNA (lncRNA), on average accounting for 18.6 to 26.9% of all detected RNA species in all sample types. In combination, the protein-coding transcripts and lncRNA on average accounted for 90.2 to 97.5% of all potentially informative RNA species detected in these sample types.

**Table 1:** Similarity of RNA species detected in ISF, serum, and plasma samples

	ISF	Serum	Plasma-1	Plasma-2
Mapped reads (M)	1.9 (1.7–4.8)	2.2 (1.3–5.6)	4.9 (2.3–6.8)	3.7 (2.5–4.8)
CDS (%)	63.2 (22.3–84.2)	70.6 (41.1–87.9)	78.9 (71.5–88.8)	71.4 (62.1–76.9)
lncRNA (%)	26.9 (7.4–74.4)	21.8 (7.1–52.3)	18.6 (7.2–26.6)	22.9 (18.8–28.0)
RNase (%)	5.4 (1.4–11.3)	3.9 (0.4–5.3)	1.2 (0.4–2.6)	2.6 (0.5–5.1)
tRNA (%)	3.5 (1.3–5.7)	2.4 (1.4–4.2)	0.7 (0.4–1.4)	1.9 (0.7–3.0)
SRP (%)	0.9 (0.2–2.6)	1.2 (0.2–3.2)	0.5 (0.3–0.7)	1.2 (0.6–1.7)
miRNA (%)	0.05 (0.01–0.09)	0.08 (0.04–0.13)	0.03 (0.01–0.05)	0.05 (0.01–0.10)
piRNA (%)	0.04 (0.00–0.07)	0.03 (0.01–0.06)	0.01 (0.00–0.01)	0.01 (0.00–0.02)

Table showing the average number of mapped reads per sample type (first row), and the percentages of those reads that mapped to RNA species of interest (average, with ranges for the four rats in parentheses), after removal of ribosomal and mitochondrial RNA hits

Of the minority RNA species, three types (RNase, tRNA, and SRP) on average accounted for 0.5 to 3.5% of all RNA species detected. Finally, miRNA and piRNA on average each accounted for less than 0.1% of all RNA species detected; it should be noted, however, that the NGS library preparation method used in this study^28^ is not well suited to analysis of short (<50 nt) RNA species, so the miRNA and piRNA levels actually present in the samples are likely higher than our measurements suggest. In any case, our results indicate that ISF, like serum and plasma, contains a wide variety of potentially informative RNA species; and that the different types of RNA present in ISF are found in roughly the same proportions in serum and plasma.

**Fig. 6:**
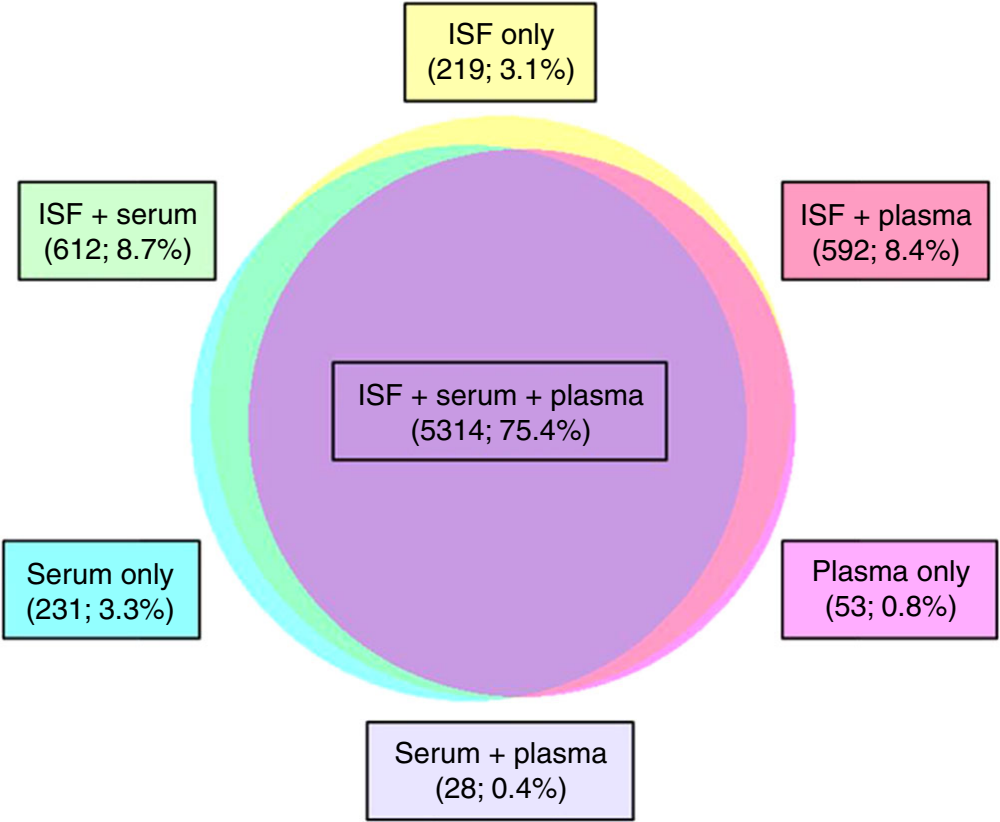
Similarity of RNA species detected in ISF, serum, and plasma samples. Proportionate Venn diagram of the 7049 protein-coding transcripts (CDS) detected in all examples of at least one sample type (e.g., all four ISF samples). Transcripts consistently detected in only one of the three sample types, as well as those detected in multiple sample types, are enumerated and expressed as a fraction of the total (7049) in boxes color-matched to the circles and their areas of overlap

Given that protein-coding transcripts comprised the majority of RNA species detected in all of the sample types analyzed, we sought to determine whether the set of transcripts consistently detected in the ISF samples resembled those consistently detected in the serum and plasma samples. For each sample type, only the transcripts detected in all examples of the type (e.g., all four ISF samples) were included in the analysis. We found that a total of 7049 transcripts were detected in all examples of at least one sample type (Fig. 6). Remarkably, 5,314 of these transcripts (75.7%) were detected in all samples analyzed, indicating that most of the transcripts consistently detected in one sample type were also consistently detected in the other sample types. Conversely, we found that few transcripts were consistently detected in one sample type but not in the others. For example, only 219 transcripts (3.1% of the total analyzed) were consistently detected in ISF but not in serum or plasma; 231 (3.3%) were consistently detected in serum only; and 53 (0.8%) were consistently detected in plasma only. In total, only 503 transcripts (7.1%) were consistently detected in only one of the three sample types analyzed. These results indicate that ISF, serum, and plasma share a core transcriptome that includes most of the transcripts consistently detected in the individual sample types. It is also interesting to note that only 312 transcripts (4.4%) were consistently detected in serum and/or plasma but not in ISF, which further suggests that the transcript content of ISF is very similar to serum and plasma.

**Fig. 7:**
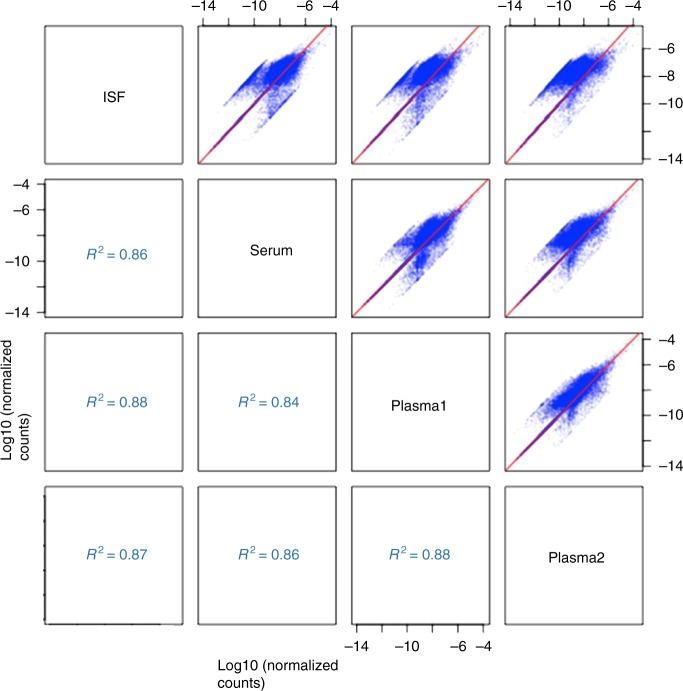
Pairwise comparison of ISF, serum, and plasma transcriptomes. Pairwise comparison of ISF, serum, and plasma transcriptomes. Four fluid samples (ISF, serum, and two plasma samples) were drawn from the same animal (Rat A), and their transcriptomes analyzed by RNA-Seq. The six panels show all the pairwise scatter plots for the transcriptomes of the four samples, where each point in blue is a gene found in both samples of the pair. Spearman Correlation Coefficient (R^2^) values for the trend/identity line in red are given

While ISF, serum, and plasma appeared similar with respect to their transcript content, we wondered whether this similarity extended to the relative abundances of their transcripts. As shown in Fig. 7, pairwise comparison of transcript abundances in ISF, serum, and plasma samples drawn from the same rat indicated close correlations in transcript abundances, such that most of the points in each scatter plot roughly followed the diagonal (marked out as a red line), and the R^2^ value (a measure of overall similarity between transcript abundances) was ≥ 0.90. Comparison of the two plasma samples yielded the highest R^2^ value (0.93), as expected; but the fact that ISF vs. serum or plasma yielded R^2^ values of 0.90, comparable to those of serum vs. plasma (0.90–0.92), underscored the similarity between ISF, serum, and plasma with respect to both transcript content and relative abundances of transcripts. Analysis of the sample sets from the other three rats supported this conclusion (Supplementary Figure 3, 4 and 5)

**Fig. 8:**
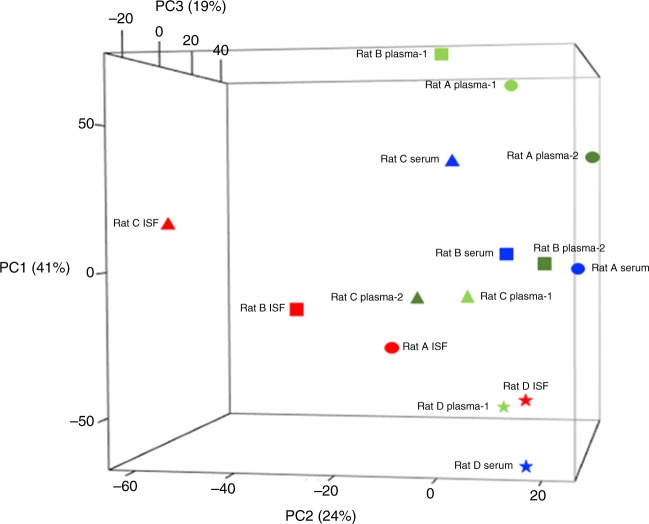
All-against-all comparison of ISF, serum, and plasma transcriptomes. Shown are principal component analysis (PCA) results that indicate the degree to which the samples’ transcriptomes (both content and relative abundances) resemble one another. Plotted points are color coded by sample type, and shaped to indicate the rat from which each sample set was drawn

All-against-all (rather than pairwise) comparison of the sample types on the basis of both transcript content and abundance, and visualization of the relationships between sample types, was accomplished through principal component analysis (PCA). While the preceding analyses highlighted the similarity between the ISF, serum, and plasma transcriptomes, the global view provided by PCA, presented in Fig. 8, suggests that the ISF transcriptome is related to, but ultimately distinct from, the serum and plasma transcriptomes, which more closely resemble one another (Fig. 6). We found that the ISF samples loosely clustered together within a horizontal plane (PC2 x PC3); the serum and plasma samples loosely clustered together within a vertical plane (PC1 x PC3); and the two planes intersected and partially overlapped, with the ISF samples from Rats A and D appearing to belong to both clusters/planes. Taken together, our results indicate that in terms of its constituents and their relative abundances, the ISF transcriptome is highly similar to serum and plasma transcriptomes, but the latter two are slightly more closely related to one another.

### Transcriptomic profiles from rats subjected to acute hypoxia

The dynamic responses of biomolecules in ISF after a physical insult are important to determine the efficacy of using dermal ISF for clinical analysis. Therefore, miRNA changes in ISF vs plasma in rats subjected to acute hypoxia was examined. Acute hypoxia affects individuals exposed to environments with frequent altitude changes, such as mountainous terrain, which can cause physiological alterations affecting pulmonary, cardiovascular, renal, neurological, and hematologic systems, as well as declines in overall performance^30–32^ and health conditions such as heart disease^32,33^ and tumor microenvironment^34,35^.

**Fig. 9:**
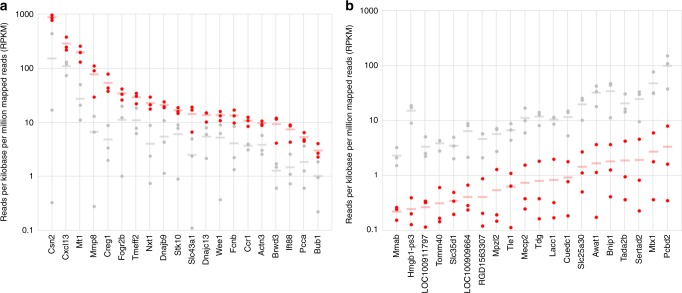
Protein-coding transcript quantification. Protein-coding transcripts enriched (**a**) and depleted (**b**) in the ISF of hypoxic (10% O2, red), as compared to normoxic (21% O2, gray), rats. The relative abundance of each transcript was measured in each of three rats per oxygen condition (six measurements per transcript in total, represented as three red and three gray data points). For each transcript, the mean of its relative abundance in the three hypoxic rats is indicated by a red bar; and the mean of its relative abundance in the three normoxic rats is indicated by a gray bar. For all of the transcripts shown, the difference in means is significant by Student’s *t*-test (*p* < 0.05)

We used our microneedle technology to extract ISF from three CD Hairless rats exposed to normoxia (21% O_2_) and three exposed to acute hypoxia (10% O_2_). We then performed RNA-Seq to identify and enumerate the transcripts present in each ISF sample. Figure 9 shows the average relative abundances of the transcripts most enriched (Fig. 9a) or depleted (Fig. 9b) in ISF collected from rats experiencing hypoxia (10% oxygen), as compared to those experiencing normoxia (21% oxygen). Previous studies have shown that matrix metalloproteinases (MMPs), as well as chemokines and their receptors, are frequently differentially expressed under conditions of hypoxia, potentially as a mechanism by which leukocyte migration is inhibited, leading to accumulation of leukocytes at hypoxic sites. Consistent with these previous studies, we found that transcripts encoding metalloproteinases (Mmp8, Mmp14/Mt1), chemokines and their receptors, (Cxcl13, Ccr1), and a known regulator of leukocyte migration (Stk10) were enriched in ISF samples collected from rats experiencing hypoxia, as compared to those collected from rats experiencing normoxia (Fig. 9a and Supplementary Table 2). Additionally, we found that transcripts encoding proteins mediating other aspects of innate immunity (Hmgb1-ps3, Lacc1, Bnip1), transcriptional regulation (Mecp2, Sertad2, Tada2b, Tle1), and mitochondrial protein import (Tomm40, Mtx1) were depleted in ISF samples collected from rats experiencing hypoxia (Fig. 7b and Supplementary Table 2). Our results indicate that hypoxia-associated changes to the ISF transcriptome can be readily detected *via* RNA-Seq, consistent with the idea that the ISF transcriptome may serve as an accessible source of molecular biomarkers that reflect changes in a subject’s physiological or disease state^36,37^.

## Conclusions

In conclusion, we describe a microneedle-based extraction method for ISF fluid in both single and microneedle array formats in both humans and rats. The technique causes minimal discomfort in human subjects and uses materials widely available to average laboratories without expensive or complicated instruments. Analysis of the extracted fluid was performed for transcriptomic, and proteomic signatures. We noted similar transcriptomic and proteomic signature profiles between ISF, serum, and plasma as well as dynamic responses of RNA-seq under hypoxia-induced conditions showing utility of ISF as a blood proxy for clinical diagnosis. Differences between ISF and blood fraction signatures noted in previous reports is likely due to localized trauma and inflammation inherent to prior ISF collection methods. We also demonstrate that dermal ISF is significantly enriched with exosomes compared to serum or plasma.

Overall, this work supports ISF sampling as a minimally invasive alternative to blood fraction analysis. It also introduces a new tool for monitoring of the dermal interstitium, allowing for further characterization of skin physiology and pathophysiology. Finally, this microneedle array prototype can be further matured and serve as the foundation for developing real-time wearable sensing technologies.

## Methods

### Microneedle fabrication

Stainless steel BD Ultra-Fine Pen needles were purchased from Becton Dickinson. The needle small diameter and five grind bevel shape are specifically engineered to limit pain and penetration force and coated with a silicone lubricant to minimize tissue trauma upon insertion^38^. Needle cap trimming was performed with a Model 6.75 60 W CO_2_ raster/vector laser system (Universal Laser Systems, Scottsdale, AZ) and position of cut was controlled through the stages and software. Repackaging the needle within the cut protective cap (Supplementary Figure 1) allowed for a controlled portion of the needle to exit the cap. Needle arrays were made by 3D printing holders (1 × 5 needle configuration) made from an acrylic resin (RGD840) with an Objet30 Pro (Stratsys) system. Single needles were placed within a PMMA holder (75 mm × 26 mm) before being applied to human subjects (Fig. 2a).

### Microneedle-assisted extraction of ISF from rats

The University of New Mexico Institutional Animal Care and Use Committee approved all experiments involving animals. Female, CD hairless Crl rats (Charles River Laboratories, Wilmington, MA), were anesthetized with 2.0% Isoflurane and 0.2 l per min oxygen. Sterile, 4 mm × 32 G, Ultra-fine Nano pen needles (BD, Franklin Lakes, NJ) were then placed into 3D-printed microneedle array holders. Furthermore, 1–5 µl calibrated pipet capillary tubes (Drummond Scientific Co., Broomall, PA) were then attached to each pen needle in the holder. The microneedle array was then gently pressed into the abdomen and/or flank dermal tissue. A small clamp was placed over the center of the holder and the array was held in place until sufficient quantity of ISF had been collected. If a decrease in ISF flow was noted, the array was removed and re-inserted in an adjacent area of the skin. In a typical experiment, moving the array multiple times allowed for collection of 30–60 µl of ISF in a 1- to 2-h period. The ISF was recovered from the capillary tubes of the microneedle array into a microcentrifuge tube on ice, an equal volume of TRIzol LS reagent (Ambion, Carlsbad, CA) was mixed in, and the sample was flash frozen using liquid nitrogen and stored at −80 °C for transcriptomic profiling.

### Microneedle-assisted extraction of ISF from human subjects

The University of New Mexico Human Research Review Committee (HRRC) approved all experiments involving human subjects. Human subjects included in the study were over the age of 18, were apparently healthy, and were not currently taking medications that affect blood clotting. Subjects with known allergy to tape and individuals with known skin disease were excluded. Informed consent was obtained from each subject prior to participation in the study. Arrays containing five needles were constructed as described above. The 3D-printed microneedle-array holders were sterilized prior to use with ethyl alcohol, and skin was cleansed with isopropyl alcohol swabs prior to array application. The microneedle array was gently pressed against the forearm with subjects in a seated or supine posture, and held in place for the duration of sample collection. Needles were held in place either by fixing with surgical wraps or by hand with no observable difference in resulting ISF volumes collected. Arrays remained in place for up to 30 min while the ISF sample was collected. The microneedle array was then withdrawn, the ISF was recovered from its capillary tubes into a microcentrifuge tube on ice, and a new array was applied for another 30 min. This cycle was repeated up to a total of six times over a maximum of a 3-h period, at which point the pooled ISF sample was flash frozen using liquid nitrogen and stored at −80 °C for proteomic profiling.

After completion of ISF collection, the subject’s forearm was inspected to make sure that no microneedle was left behind in the skin, and the skin was again cleansed with an alcohol swab. At the beginning, middle, and end of the sample collection, each subject was asked whether any pain or discomfort was experienced. If there was pain or discomfort, the subject was asked to rate it on a scale from 1–10, with 1 being mild irritation and 10 being severe pain.

### Blood fraction collection for transcriptomic and proteomic profiling

Following completion of ISF collection from a rat, terminal blood was collected, and the animal euthanized, via cardiac puncture. The blood sample was split equally between a Vacutainer serum tube and a Vacutainer plasma tube (BD, Franklin Lakes, NJ), both of which were centrifuged at 3,000 x g for 15 min. The serum and plasma fractions were recovered into individual microcentrifuge tubes, mixed with an equal volume of TRIzol LS reagent (Ambion, Carlsbad, CA), flash frozen using liquid nitrogen, and stored at −80 °C for transcriptomic profiling.

Prior to ISF collection from a human subject, blood was collected via venipuncture, and the sample split equally between a Vacutainer serum tube and a Vacutainer plasma tube (BD, Franklin Lakes, NJ), both of which were centrifuged at 3,000 x g for 15 min. The serum and plasma fractions were recovered into individual microcentrifuge tubes, flash frozen using liquid nitrogen, and stored at −80 °C for proteomic profiling.

### Exosome isolation, TEM, and protein quantification

Exosomes were purified from ISF and serum according to manufacturer protocols using a Total Isolation Kit from Other Fluids and a Total Isolation Kit from Serum (Invitrogen, Carlsbad, CA), respectively. Exosomes were visualized using TEM. The 5 µl drops of each purified exosome suspension were applied to carbon-coated grids and allowed to dry for 5 min. Furthermore, 5 µl of a 4% uranyl acetate solution was then added to the grids to negatively stain the grid. The solution was allowed to dry on the grid for 5 min. The samples were then imaged using a Hitachi 7700 TEM with an acceleration voltage of 80 kV. Exosome diameters were calculated using ImageJ Software. Between 30 and 80 exosomes were counted and the mean Feret’s diameters and standard deviations were calculated. To determine the amount of total protein in each purified exosome preparation (protein equivalent), a nanoDrop spectrophotometer was set to measure at 280 nm and first blanked with the PBS buffer. Absorbance values of 1.0 µl drops of each exosome purification product were then measured at 280 nm.

### Sample preparation for ISF transcriptomic profiling of ISF

An ISF sample, a serum sample, and two plasma samples were collected from each of four rats (with only one plasma sample collected from the fourth rat). For each sample, total RNA was extracted, rRNA content was depleted through molecular normalization, and the remaining RNA species analyzed through NGS (i.e., RNA-Seq). Sequencing reads that mapped to annotated genome features were enumerated, and the counts normalized for sampling depth and transcript length, as described below.

RNA was extracted from rat ISF, serum, and plasma samples using a previously described method.In brief, each sample (ISF, serum, or plasma mixed 1:1 with TRIzol LS reagent and stored at −80 °C) was thawed and then centrifuged at 16,000 rpm at 4 °C for 15 min. The aqueous phase was transferred to a new tube and mixed with an equal volume of 100% ethanol, and total RNA extracted from the mixture using the Direct-zol RNA Miniprep Kit (Zymo Research, Irvine, CA, USA), following the manufacturer’s instructions. Total RNA was eluted in 10 µl sterile nuclease-free water.

Synthesis of cDNA flanked with appropriate adapters and barcodes for Next Generation Sequencing (NGS) was carried out using the Peregrine method, as previously described^28^.

Molecular normalization of double-stranded cDNA products [to selectively deplete highly abundant cDNA species, primarily those derived from ribosomal RNA (rRNA)] was carried out using hydroxyapatite chromatography (HAC)^39^ implemented in microcentrifuge tube format (spin-HAC), as follows. Each cDNA sample was diluted to 10 ng per µl in 4X hybridization buffer (200 mM HEPES, pH 7.5, 2 M NaCl) in a total volume of 10 µl, then incubated at 98 °C for 3 min (to denature the cDNA) followed by 68 °C for 5 h (to allow re-annealing of highly abundant cDNA species). To prepare the spin-HAC column, BioGel HTP DNA-grade hydroxyapatite medium (BioRad, Hercules, CA, USA) was hydrated with 10 mM sodium phosphate (pH 7.0) + 20% formamide (Buffer A); 100 µl of the hydrated gel was loaded into a Pierce Micro-Spin Column (Thermo Fisher Scientific, Waltham, MA, USA); and the column was washed twice with Buffer A at 60 °C. The re-annealed cDNA sample was loaded onto the prepared column and allowed to interact with the binding matrix at 60 °C for 5 min. Finally, after washing the column twice with Buffer A at 60 °C, the single-stranded cDNA species were selectively eluted using pre-warmed 100 mM sodium phosphate (pH 7.0) + 20% formamide (Buffer B). The eluted cDNA species were purified using 1.6X volumes of Agencourt AMpure XP beads (Beckman Coulter Life Sciences, Indianapolis, IN, USA), recovered into 25 μl of nuclease-free water, and the concentration measured by qPCR as previously described^33^.

Four to six cDNA libraries bearing different barcodes were mixed in equal molar ratios, the multiplexed library further concentrated using the Zymo DNA Clean and Concentrator-5 system (Zymo Research, Irvine, CA, USA), and its final concentration measured using the Kapa qPCR assay (Kapa Biosystems, Wilmington, MA, USA). The multiplexed library was then loaded onto a NextSeq 500 (Illumina, San Diego, CA, USA) for a 75 nt single-end run.

### Bioinformatics analyses of sequencing results

Demultiplexed read sets were quality-filtered and primer-trimmed using bbduk 35 (http://sourceforge.net/projects/bbmap/) with parameters ktrim = *r* *k* = 21 mink = 11 hdist = 1. Reads were matched to rat rRNA and mitochondrial RNA sequences using bowtie2^40,41^ with these parameters -D 20 -R 3 -N 1 -L 20 -i S,1,0.50 --local. Remaining reads were mapped to the rat genome 6.0, using bowtie2 as above except in end-to-end mode. Mapping to genes was performed using htseq-count 0.5.3^40^ in intersection-strict mode, using the annotation file from NCBI, and summing all CDS counts for each protein-coding gene.

Read count data were used for correlation and principal component analysis (PCA) as follows. First, a count value cutoff of 10 was imposed to remove low-count data (i.e., only genes receiving ≥ 10 counts in ≥ 1 sample were included in downstream analyses). CDS count data surpassing this threshold were normalized for total number of counts per sample, as well as for the length of the CDS as measured in base pairs (generating an RPKM-like value). The samples were batch normalized using an empirical Bayes method^42^, based on sample source metadata. For PCA, we used the prcomp function in R, the data were mean-variance scaled, and the results were plotted in 3D (PC1 vs. PC2 vs. PC3). For correlation scatter plots, the data were log-transformed before plotting, and Spearman Correlation R-squared values were calculated.

### The 48-h hypoxia procedure

Three to seven-month-old, female, CD hairless Crl rats, obtained from Charles River Laboratories (Wilmington, MA), were placed into standard rat cages equipped with lids, cut to size, from Plexiglas. The Plexiglas had four 1 ½ cm holes drilled in the top. One hole was used to hold the water bottle. Two holes were kept open to allow carbon dioxide to escape and the last hole was used to insert tubing, which carried gas mixtures into the cage. A gas mixing box was used to control the oxygen levels being delivered to the cages. Four groups of rats were exposed to 48 h of either 21% O2/79% N2 (normoxia) or 10% O2/90% N2 (hypoxia). Oxygen levels in the cages were checked several times a day using an oxigraph, in order to ensure that the appropriate levels were maintained throughout the exposure period. At the end of 48 h, ISF samples were collected from the rats using the five-microneedle array described above, while normoxia vs. hypoxia was maintained through supply of the appropriate gas mixture (21% O2/79% N2 vs. 10% O2/85% N2) via nose cone. Using this approach, ISF samples were collected in volumes ranging from 35 to 60 µl per rat.

### Sample processing for proteomic profiling

ISF, plasma, and serum samples were extracted from three rats and preserved as described above^43^. After thawing the samples, they were subjected to reduction, alkylation, tryptic digestion, tandem mass tagged (TMT) labeling, and two-dimensional separation prior to mass spectrometry acquisition. Protein concentration was measured using Pierce BCA protein assay kit following the manufacturer’s protocol. An aliquot of 135 µg of total protein was added in 500 µl of a buffer of 20 mM triethylammonium bicarbonate (TEAB) (Sigma-Aldrich, prod # T-7408) and 6 M urea. Protein reduction was performed by adding 10 µl of 1 M dithiothreitol (Sigma-Aldrich, prod # D0632–25G) for a 30-min incubation at 56 °C on shaker at 400 rpm. Alkylation of cysteine was carried out with addition of 40 µl of 0.5 M iodoacetamide (Sigma-Aldrich, St. Louis, MO; cat# I1149–25G) at room temperature for 30 min in the dark. The alkylated samples were diluted to a final urea concentration of 2 M with 50 mM TEAB solution, and 4 µg Lys-C/Trypsin (Promega, prod # V5071) was added for digestion overnight at 37 °C on shaker at 400 rpm. Tryptic digests were acidified to 1% formic acid and cleaned up on 1cc Oasis HLB C18 SPE cartridges (Waters, WAT094225) following the manufacturer’s protocol. Peptide eluents were speedvac to dryness and then re-suspended in 112.5 µl of 10% acetonitrile/0.1 M TEAB. An aliquot of 56 µl (33.75 µg) each sample was labeled with 10-plex TMT isobaric reagents (Thermo Fisher Scientific, Waltham, MA; cat# 90110) following the manufacturer’s protocol. The labelled samples were combined and speedvac to dryness.

### Basic reverse phase peptide fractionation

TMT labelled samples were fractionated in basic reverse phase condition as previously described^43^. Briefly, the pooled peptides re-suspended in 1.5 ml of mobile phase A of 10% acetonitrile/100 mM ammonium formate pH 10 were loaded on a C18 trap column at flow rate of 0.2 ml per min. Separation of trapped peptides were carried out on a 4.6 mm × 250 mm, 5 µm XBridge column (Waters) at flow rate of 0.5 ml/min using a gradient profile of 0–5 min: 0% B, 5–13 min: 0 to 15% B, 13–46 mins: 15 to 28.5% B, 46–51.5 mins: 28.5 to 34% B, and 51.5–64.5 mins: 34 to 60% B. Eluent was collected in four ‘beginning’ fractions of 2 min^44^, then 84 fractions of 0.5 min, and 5 ‘end’ fractions of 2 min. Both the beginning and end fractions were pooled into two separate fractions. The 84 fractions were concatenated into 28 fractions making for a total of 30 fractions to be analyzed. Each fraction was speedvac to dryness and stored at −80 °C until mass spectrometric analysis.

### Liquid chromatography mass spectrometry analysis (LC-MS/MS)

Each pH 10 fraction was re-suspended in 20 µl of acetonitrile/water/formic acid 5/95/0.5 (v/v/v) and 2 µl was analyzed in a nanoLC system (Dionex, Ultimate 3000) coupled to a Q Exactive plus mass spectrometer (Thermo Fisher Scientific, Waltham, MA). Peptides were loaded on a PepMap trap 300 um x 5 mm C18, 100 A (Thermo Fisher Scientific, Waltham, MA) at flow rate of 10 µl per min for 5 min. Peptides were then separated on an EASYspray C18 75 µm x 50 cm analytical column over a 182-min run at flow rate of 300 nl/min using mobile phase A of 0.1% formic acid in water and mobile phase B of acetonitrile/water/formic acid 80/20/0.1 (v/v/v). LC gradient was 0–150 min: 6–35%B, 150–158 min: 35–60%B, 158–161 min: 60–90%B, 161–171 min: 90% B hold, 171–172 min: 90–6% B, and 172–182 min: 6% B hold.

Mass spectrometric data was acquired using a Top12 data-dependent method. Full MS scans were measured at a resolving power 70,000 over an *m*/*z* range 300–1700. Monoisotopic peak of top 12 most abundance ions with charge states 2–7 in each MS scan were isolated with isolation window of 2 m/z for higher energy collisional dissociation (HCD) at normalized collision energy of 26. MS2 spectrum started at m/z 100 was measured at a resolving power of 35,000. Dynamic exclusion time window of 20 s was set to filter the acquired precursor ions.

### Proteomics data processing and bioinformatics analysis

MS data was searched against a UniprotKB *Rattus norvegicus* database using software Proteome Discoverer 2.1 SP1 (Thermo Fisher Scientific, Waltham, MA). Searching parameters were set to a precursor mass tolerance 10 ppm, fragment tolerance 0.02 Da, maximum missed cleavage level of 2, minimum peptide length of 6, variable modifications of oxidized methionine [+15.99 Da] and acetylated N-protein terminus [ + 42.011 Da] were set as variable modifications, and carbamidomethylation of cysteine [ + 57.02 Da], N-terminal TMT 6 plex [ + 229.16 Da] and TMT labeling of lysine [ + 229.16 Da] as static modifications. High confidence peptide spectrum matches (PSM) filtered at a false discovery rate of 1% using Percolator algorithm^45^ were applied to peptide identification. Peptide identification and protein identification were set at high confidence level with a false discovery rate of 1%. One protein is determined when having at least two corresponding identified peptides and 1 peptide unique to the protein group.

Reporter ion intensities of unique and razor peptides were extracted from MS2 spectra at mass tolerance of 20 ppm and normalized to total peptide amount of the highest channel within the LC-MS/MS run. The normalized intensities were scaled to 100 for calculating protein relative abundance ratios and imported to Perseus 1.5.5.3^46^ for statistical analysis of protein abundances among the experimental samples. Annotation enrichment analysis were performed using DAVID 6.8^47^.

## Electronic supplementary material

Supplementary Information

## Data Availability

The mass spectrometry proteomics data have been deposited to the ProteomeXchange Consortium via the PRIDE^48^ partner repository with the dataset identifier PXD010879. Transcriptomic data are available via the NCBI Sequence Read Archive (SRA) with the BioProject identifier PRJNA489285.
