# Supplementary material for: Extraction and biomolecular analysis of dermal interstitial fluid collected with hollow microneedles
Source: Commun Biol. 2018 Oct 22;1:173. doi: 10.1038/s42003-018-0170-z (PMC6197253; doi:10.1038/s42003-018-0170-z)
Supplement: Supplementary file 1 — Supplementary Information [file 42003_2018_170_MOESM1_ESM.docx]

**Supplementary Figures**


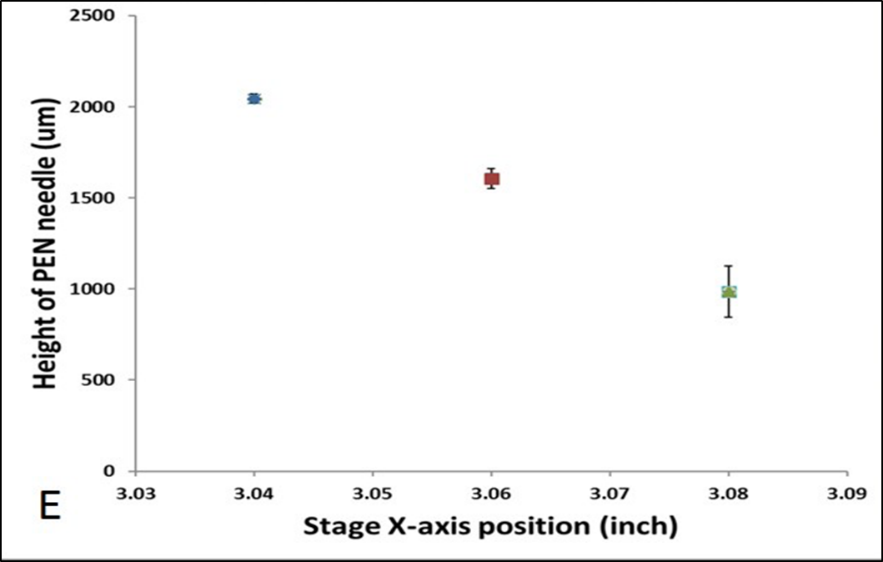


**Supplementary Figure 1.** Laser cutting position of the stage on original needle holders to control needle final length protruding out of resulting cut holder.


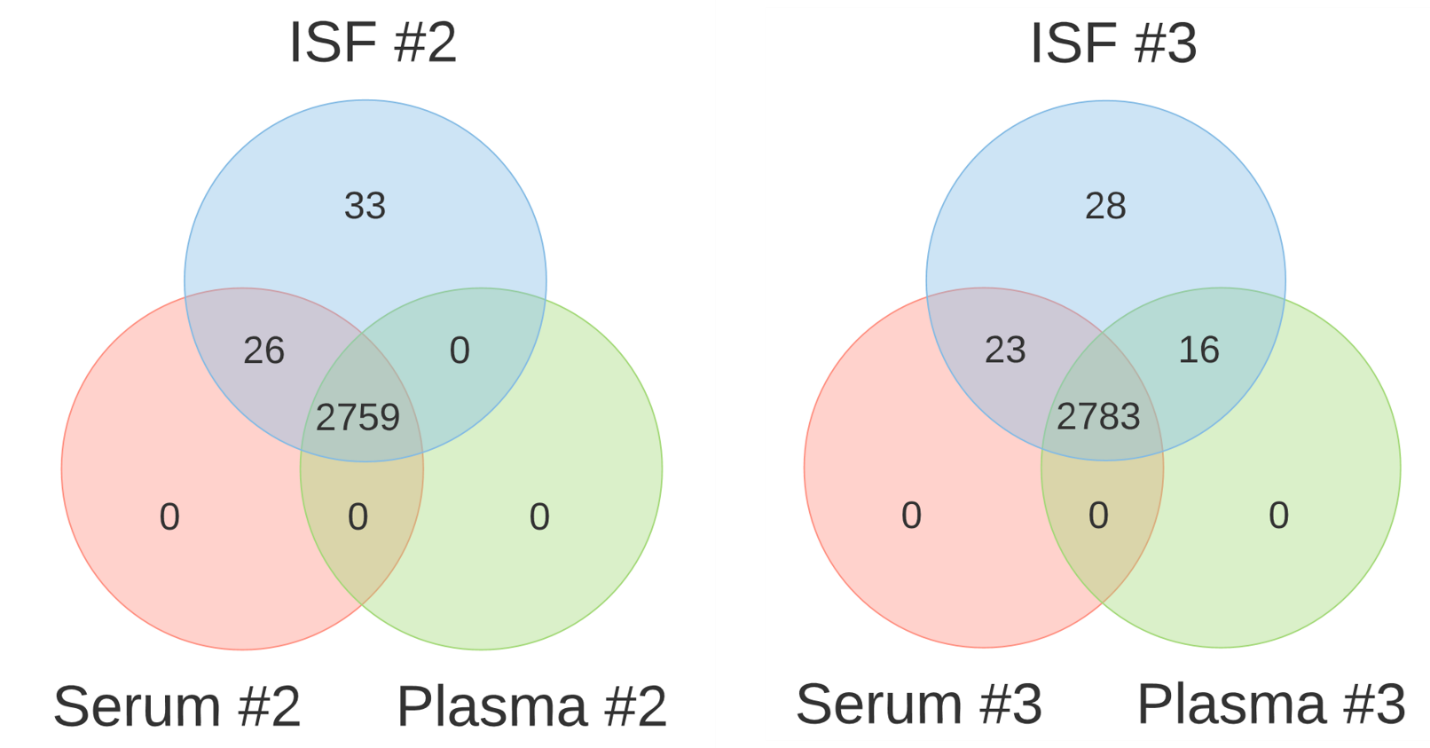


**Supplementary Figure 2.** Total identified proteins and their distribution in plasma, serum and ISF of rats #2 and 3.


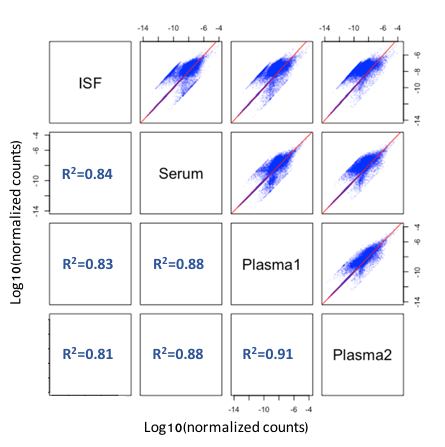


**Supplementary Figure 3. Pairwise comparison of ISF, serum, and plasma transcriptomes: Rat B samples.** One ISF, one serum, and two plasma samples were drawn from the same animal (Rat B), and their transcriptomes analyzed by RNA-Seq. Pairwise comparison of the transcriptomes, with regard to the relative abundances of transcripts held in common, is visualized in scatter plot format (upper right), and the degree to which they correlate expressed as a Spearman Correlation Coefficient (R^2^) value (lower left).


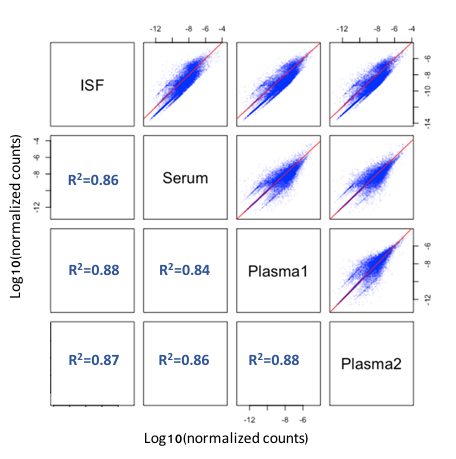


**Supplementary Figure 4. Pairwise comparison of ISF, serum, and plasma transcriptomes: Rat C samples.** One ISF, one serum, and two plasma samples were drawn from the same animal (Rat C), and their transcriptomes analyzed by RNA-Seq. Pairwise comparison of the transcriptomes, with regard to the relative abundances of transcripts held in common, is visualized in scatter plot format (upper right), and the degree to which they correlate expressed as a Spearman Correlation Coefficient (R^2^) value (lower left).


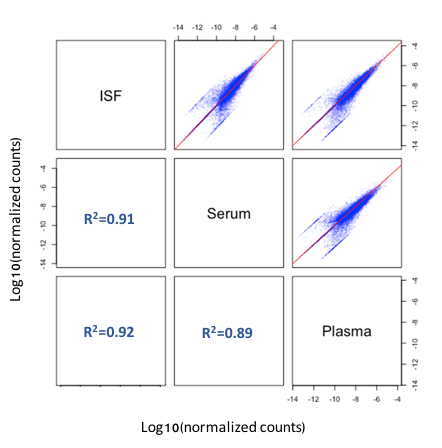


**Supplementary Figure 5. Pairwise comparison of ISF, serum, and plasma transcriptomes: Rat D samples.** ISF, serum, and plasma samples (one each) were drawn from the same animal (Rat D), and their transcriptomes analyzed by RNA-Seq. Pairwise comparison of the transcriptomes, with regard to the relative abundances of transcripts held in common, is visualized in scatter plot format (upper right), and the degree to which they correlate expressed as a Spearman Correlation Coefficient (R^2^) value (lower left).

**Supplementary Tables**

| **Category** | **Term** | **Count** | **%** | **P-value** |
| --- | --- | --- | --- | --- |
| CC_DIRECT | extracellular exosome | 194 | 69.5 | 1.30E-100 |
| CC_DIRECT | cytosol | 105 | 37.6 | 1.60E-41 |
| CC_DIRECT | cytoplasm | 173 | 62 | 3.50E-33 |
| CC_DIRECT | myelin sheath | 27 | 9.7 | 7.10E-18 |
| CC_DIRECT | cell-cell adherens junction | 29 | 10.4 | 1.70E-16 |
| BP_DIRECT | cell-cell adhesion | 27 | 9.7 | 1.80E-16 |
| BP_DIRECT | glycolytic process | 10 | 3.6 | 5.60E-09 |
| BP_DIRECT | gluconeogenesis | 8 | 2.9 | 5.50E-07 |
| BP_DIRECT | glutathione metabolic process | 9 | 3.2 | 1.10E-06 |
| BP_DIRECT | response to drug | 23 | 8.2 | 2.20E-05 |
| MF_DIRECT | cadherin binding involved in cell-cell adhesion | 29 | 10.4 | 3.30E-17 |
| MF_DIRECT | protein binding | 66 | 23.7 | 2.90E-12 |
| MF_DIRECT | protein homodimerization activity | 39 | 14 | 4.60E-09 |
| MF_DIRECT | GTP binding | 23 | 8.2 | 3.50E-07 |
| MF_DIRECT | actin filament binding | 14 | 5 | 5.00E-07 |

**Supplementary Table 1:** Functional annotation analysis demonstrating top 5 enriched terms per categories of cellular component, biological process and molecular function of the 301 proteins (279 recognized genes) that were ANOVA significantly changed and higher expressed in ISF.

| **Function** | **Genes** |
| --- | --- |
| **Matrix Metalloproteinases (MMPs)** | **Mmp8, Mt1** |
| **Chemokines** | **Cxcl13, Ccr1** |
| **Leukocyte Migration** | **Stk10** |
| **Innate Immunity** | **Hmgb1-ps3, Lacc1, Bnip1** |
| **Transcription Regulator** | **Creg1, Brwd3, Mecp2, Sertad2, Tada2b, Tle1** |
| **Mitochondrial Processes** | **Pcca, Tomm40, Mtx1** |
| **Heat Shock Protein Regulation** | **Dnajb9** |
| **Cytoskeleton and Cell Adhesion** | **Mpzl2, Actn3** |
| **DNA Repair** | **Tdg** |

**Supplementary Table 2. Annotated functions of genes encoding transcripts that are enriched (red) or depleted (blue) in the ISF of rats experiencing hypoxia (10% oxygen), as compared to rats experiencing normoxia (21% oxygen).**
